# Volume overload impedes the maturation of sarcomeres and T-tubules in the right atria: a potential cause of atrial arrhythmia following delayed atrial septal defect closure

**DOI:** 10.3389/fphys.2023.1237187

**Published:** 2023-10-16

**Authors:** Zhuoya Dong, Dian Chen, Sixie Zheng, Zheng Wang, Debao Li, Yingying Xiao, Sijuan Sun, Lincai Ye, Lisheng Qiu, Yuqing Hu, Haifa Hong

**Affiliations:** ^1^ Department of Pediatric Intensive Care Unit, Ningbo Women and Children’s Hospital, Ningbo, Zhejiang, China; ^2^ Department of Thoracic and Cardiovascular Surgery, Shanghai Children’s Medical Center, School of Medicine, Shanghai Jiao Tong University, Shanghai, China; ^3^ Department of Thoracic and Cardiovascular Surgery, Shanghai Children’s Hospital, School of Medicine, Shanghai Jiao Tong University, Shanghai, China; ^4^ Department of Pediatric Intensive Care Unit, Shanghai Children’s Medical Center, School of Medicine, Shanghai Jiao Tong University, Shanghai, China; ^5^ Institute of Pediatric Translational Medicine, Shanghai Children’s Medical Center, School of Medicine, Shanghai Jiao Tong University, Shanghai, China; ^6^ Shanghai Institute for Pediatric Congenital Heart Disease, Shanghai Children’s Medical Center, School of Medicine, Shanghai Jiao Tong University, Shanghai, China; ^7^ Department of Cardiology, Shanghai Children’s Medical Center, School of Medicine, Shanghai Jiao Tong University, Shanghai, China

**Keywords:** volume overload, atrial septal defects, atrium, maturation, sarcomere, transverse tubules

## Abstract

**Introduction:** Adult patients with atrial septal defects (ASD), the most common form of adult congenital heart disease, often die of arrhythmias, and the immaturity of cardiomyocytes contributes significantly to arrhythmias. ASD typically induces a left-to-right shunt, which then leads to the right atrium (RA) volume overload (VO). Whether or not VO contributes to RA cardiomyocyte immaturity and thereby causes arrhythmias in adult patients with ASD remains unclear.

**Methods:** Here, we developed the first neonatal RA VO mouse model by creating a fistula between the inferior vena cava and abdominal aorta on postnatal day 7. RA VO was confirmed by increases in the mean flow velocity, mean pressure gradient, and velocity time integral across the tricuspid valve, and an increase in the RA diameter and RA area middle section.

**Results:** We found that VO decreased the regularity and length of sarcomeres, and decreased the T-element density, regularity, and index of integrity of T-tubules in RA cardiomyocytes, suggesting that the two most important maturation hallmarks (sarcomere and T-tubules) of RA cardiomyocytes were impaired by VO. Accordingly, the calcium handling capacity of cardiomyocytes from postnatal day 21 (P21) RA was decreased by VO. VO caused a significant elongation of the PR interval. The expression of connexin 43 (Cx43) was decreased in RA VO. Moreover, gene ontology (GO) analysis of the downregulated genes in RA demonstrated that there was an abundance of enriched terms associated with sarcomeres and T-tubules exposed to VO. The results were further verified by qRT-PCR.

**Conclusions:** In conclusion, the first neonatal RA VO mouse model was developed; furthermore, using this neonatal RA VO mouse model, we revealed that VO impeded RA sarcomere and T-tubule maturation, which may be the underlying causes of atrial arrhythmias in adult patients with ASD.

## 1 Introduction

Isolated atrial septal defect (ASD) occurs in approximately 2/1,000 live births and is the most common form of adult congenital heart disease (CHD) (Stout et al., 2018; Brida et al., 2022). This is because more than 97% of children born with ASD live to adulthood (Stout et al., 2018; Brida et al., 2022). However, adult patients with ASD present with various types of complications, such as right heart failure, pulmonary hypertension, thrombosis, arrhythmias, and even sudden death (Nitta et al., 2013; Himelfarb et al., 2022), and its underlying mechanisms remain elusive. Thus, current ASD guidelines recommend that ASD closure should be based on the severity of a patient’s clinical symptoms and age (Stout et al., 2018).

The most important hemodynamic feature of ASD is a left-to-right shunt at the atrial level, resulting in volume overload (VO) in the right atrium (RA) (Lindsey and Hillis, 2007; Humenberger et al., 2011). Clinical retrospective studies have found that patients in whom the ASD is closed before the onset of arrhythmias have a lower rate of subsequent recurrence of arrhythmias than those whose ASD is closed after the onset of arrhythmias; however, its underlying mechanisms are not clear (Silversides et al., 2004; Humenberger et al., 2011; Rigatelli et al., 2021). More importantly, when ASD is closed in adulthood, the altered electrophysiological function is not restored, with the most prominent abnormalities being atrial fibrillation and atrial flutter (Jategaonkar et al., 2009; Oliva et al., 2022; Muroke et al., 2023). Previous studies speculated that this may be due to irreversible RA structural and electrophysiological damage caused by VO (Silversides et al., 2004; Rigatelli et al., 2021). However, the type of damage and the underlying mechanisms are unclear.

During the postnatal cardiac developmental process, cardiomyocytes undergo a maturation transformation to meet the physiological needs of the systemic circulation, which mainly includes the following: 1) sarcomere maturation, in which the sarcomere components change from MYH7 to MYH6, from TNNI1 to TNNI3, and from a disordered and irregular sarcomere arrangement to a rod-like and ordered sarcomere arrangement (Schiaffino et al., 1993; Opitz et al., 2004; Bray et al., 2008); 2) electrophysiological maturation, in which the transverse tubules (T-tubules) gradually increase in density and integrity, with an increased calcium-handling ability (Perera et al., 2022; Smith et al., 2022; Zhang et al., 2022; Tarasov et al., 2023). Cardiomyocyte maturation failure may lead to a range of clinical symptoms, including arrhythmias and heart failure (Campostrini et al., 2021; Funakoshi et al., 2021; Dimasi et al., 2023; Lou et al., 2023). Whether VO impedes RA cardiomyocyte maturation and thereby causes atrial arrhythmias in adult patients with ASD is unclear.

One of the reasons why this phenomenon is not well understood is the lack of a neonatal RA VO mouse model. There are two challenges in creating a neonatal RA VO mouse model. First, the method for neonatal anesthesia is ice cooling, which requires that the operation time on the neonatal mice should not exceed 15 min (Cui et al., 2021; Zhou et al., 2022). Second, the extremely small size of neonatal mouse hearts requires advanced microsurgical skills (Sun et al., 2021; Hu et al., 2022). In the past, cardiac VO models were reported only in adult mice or large animals, and there were no neonatal mouse cardiac VO models (Sun et al., 2019; Cui et al., 2021; Sun et al., 2021; Zhou et al., 2021; Hu et al., 2022; Zhou et al., 2022). Recently, we used neonatal aortocaval fistula (ACF) surgery to increase the amount of blood returning to the heart (Sun et al., 2019; Cui et al., 2021; Sun et al., 2021; Zhou et al., 2021; Hu et al., 2022; Zhou et al., 2022), as ACF has been confirmed to produce ventricular VO (Sun et al., 2019; Cui et al., 2021; Sun et al., 2021; Zhou et al., 2021; Hu et al., 2022; Zhou et al., 2022). In theory, increased blood returning to the heart by ACF could also cause RA VO, but experimental data were needed to support the theory.

In the present study, we first established a neonatal RA VO mouse model by ACF surgery and determined that ACF induced neonatal RA VO in mice. We subsequently applied this model to investigate the impact of RA VO on cardiac arrhythmia. Our investigation revealed that RA cardiomyocytes exposed to VO exhibited reduced sarcomere lengths and disrupted regularity, indicative of impaired sarcomere maturation within RA. Furthermore, VO was associated with a diminished density, disrupted regularity, and compromised integrity of T-tubules, leading to a decreased amplitude of calcium transients and an extended time to peak. The T-tubule system serves as the structural foundation for the excitation–contraction coupling of cardiomyocytes and is crucial for their calcium-handling capabilities. Anomalies in calcium handling are closely associated with the occurrence of cardiac arrhythmias. Moreover, we analyzed atrial electrophysiological characteristics using electrocardiographic measurements and observed a significant prolongation of PR intervals in mice subjected to VO. This alteration in PR intervals can elevate susceptibility to atrial arrhythmias. Furthermore, we examined other arrhythmogenic substrates and observed a significant lateralization of connexin 43 (Cx43), accompanied by a marked reduction in the percentage of Cx43 in the intercalated discs under VO conditions. These findings provide novel insights into the connections between RA VO and the occurrence of atrial arrhythmias. This study provides a theoretical basis to prevent arrhythmia in adult patients with ASD and is a pioneering study of neonatal RA VO.

## 2 Materials and methods

The data generated in this study are available from the corresponding author upon reasonable request. All RNA-seq data have been deposited in the GEO database (https://www.ncbi.nlm.nih.gov/geo), with accession number GSE232594.

All of the primer and reagent information is provided in Supplementary Tables S1, S2.

### 2.1 ACF surgery

C57BL/6 neonatal mice, male or female, were randomized into two groups, ACF and sham surgery, at postnatal day 7 (P7), as described in previous publications (Stout et al., 2018; Brida et al., 2022). In brief, under general anesthesia (4%–5% isoflurane), a midline laparotomy was performed on the pups to expose the abdominal aorta (AA) and inferior vena cava (IVC). ACF was created by a puncture through AA into IVC with a needle (diameter, 0.08 mm). Then, the abdominal wall was closed with local lidocaine treatment to relieve pain.

### 2.2 Ultrasonography

The ACF and tricuspid valve flow were analyzed using a Vevo 3100 Imaging System (VisualSonics, Toronto, Ontario, Canada) with a pulse-wave mode, as described in previous publications (Sun et al., 2021; Hu et al., 2022).

### 2.3 Histology and immunohistochemistry

Hearts were dewatered, embedded in paraffin, sliced (8 μm thickness), and stained with hematoxylin and eosin (H&E), Sirius Red, and Masson’s trichrome according to the manufacturer’s instructions (G1120, G1340, and G1472; Solarbio Life Science, Beijing, China). Atrial expression and distribution of Cx43 (CST, #3512) were studied using an immunohistochemistry (IHC) method. Sections were incubated with an HRP antibody for 30 min at 37°C. A DAB chromogenic reagent kit (G1212, Servicebio Technology Co., Ltd., Wuhan, China) was used for coloration. Quantification was calculated using Fiji 2.9.0 (National Institutes of Health, United States).

### 2.4 Hemodynamic measurement

We conducted hemodynamic measurements on both sham and VO mice at P56. We utilized pressure transducers and the PowerLab system (ADInstruments, Colorado Springs, CO) to measure and record end-systolic (RVSP), end-diastolic (RVDP), and mean (mRVP) pressures of the right ventricle.

### 2.5 RNA extraction and qRT-PCR

After anesthetizing with 1.5% isoflurane, mice at P21 were euthanized to obtain the RAs, which were used for subsequent experiments. RNA extraction was performed using the PureLink RNA Micro Scale Kit. RNA integrity was evaluated using the RNA Nano 6000 Assay Kit of the Bioanalyzer 2100 system (Agilent Technologies, CA, United States). Quantitative real-time PCR (qRT-PCR) was performed using SYBR Green Power Premix kits according to the manufacturer’s instructions. The primers were obtained from Generay Biotech Co., Ltd. (Shanghai, China).

### 2.6 Library preparation

The sequencing libraries were generated using the NEBNext^®^ Ultra^TM^ RNA Library Prep Kit for Illumina^®^ (NEB, United States) according to the manufacturer’s instructions. The library fragments were purified using an AMPure XP system (Beckman Coulter, Beverly, MA, United States), and library quality was assessed on the Agilent Bioanalyzer 2100 system.

### 2.7 Clustering, sequencing, and mapping

The clustering of the index-coded samples was performed on a cBot Cluster Generation System using a TruSeq PE Cluster Kit v3-cBot-HS (Illumina). Sequencing was performed on an Illumina NovaSeq platform to generate 150-bp paired-end reads. Raw data (raw reads) in the FASTQ format were processed through in-house Perl scripts to generate clean data (clean reads). All of the downstream analyses were thus based on clean, high-quality data. The indexes of the reference genome and paired-end clean reads were constructed using HISAT2 v2.0.5. The number of reads mapped to each gene was counted using featureCounts v1.5.0-p3. The fragments per kilobase of transcript sequence per million base pairs sequenced (FPKM) for each gene was calculated based on the length of the gene and read counts mapped to each gene.

### 2.8 Differential gene expression analysis

Differential gene expression was analyzed using the DESeq2 R package (1.16.1). Genes with an adjusted *p*-value of <0.05, as determined using DESeq2, were considered to be differentially expressed.

### 2.9 GO enrichment analyses

GO enrichment analysis of downregulated genes was implemented using the clusterProfiler R package. GO terms with corrected *p* < 0.05 were considered to be significantly enriched.

### 2.10 T-tubule imaging

*In situ* T-tubule imaging and AutoTT analysis were performed as described previously (Sun et al., 2021; Hu et al., 2022). Intact mice hearts were Langendorff-perfused with Tyrode’s solution containing 2.5 μM of FM 4-64 (Invitrogen™, Paisley, UK) for 20 min. The hearts were placed in the perfusion chamber attached to the stage of a confocal microscope and perfused with the indicator-free/Ca^2+^-free solution. The membrane structure of epicardial myocytes was analyzed *in situ* using a confocal microscope with a ×63 oil immersion lens. AutoTT preprocessed the confocal images and then extracted and analyzed T-tubule system morphological features.

### 2.11 Sarcomere imaging

Sarcomere imaging was performed as described in previous publications (Sun et al., 2021; Hu et al., 2022). RA cardiomyocytes were isolated using a Langendorff perfusion system, then fixed with 4% paraformaldehyde for 10 min, permeated with 0.5% Triton X-100 for 15 min, stained with sarcomeric α-actinin (SAA, 1:200 dilution, Abcam) overnight at 4°C, imaged using a confocal microscope with a ×60 objective, and finally analyzed using AutoTT.

### 2.12 Cardiomyocyte isolation and calcium imaging

Cardiomyocytes were isolated using a Langendorff perfusion system, as described previously (Sun et al., 2021). After perfusion, only RA was removed, and cardiomyocytes from the RA were used for calcium imaging. Calcium imaging was performed according to a previous publication (Sun et al., 2021). In brief, before contractility and calcium analyses, calcium was re-introduced into isolated cardiomyocytes by treating the cells with a series of 10 mL of 2,3-butanedione monoxime-free perfusion buffers containing 100 nmol/L, 400 nmol/L, 900 nmol/L, and 1.2 μmol/L CaCl_2_. At each step, cardiomyocytes were settled down by gravity for 10 min at room temperature before cells were transferred to the next buffer with a higher calcium concentration. Cardiomyocytes were loaded with 5 μmol/L Rhod-4™, AM (21121, AAT Bioquest, United States) for 20 min. Then, the cells were washed with normal Tyrode’s solution (NaCl, 140 mmol/L; KCl, 4 mmol/L; MgCl_2_, 1 mmol/L; CaCl_2_, 1.8 mmol/L; glucose, 10 mmol/L; and HEPES, 5 mmol/L, pH = 7.4, adjusted with NaOH) for 20 min. The cells were subsequently settled in a laminin-coated glass-bottomed flow chamber at 30°C for 10 min and electrically stimulated at 1 Hz to produce steady-state conditions. Finally, calcium signals were acquired through confocal line scanning using a ×63 objective. A line scan was positioned along the long axis of the cell in the cytosol, avoiding the nuclear area. Calcium signals were quantified manually using Fiji 2.9.0.

### 2.13 Electrocardiogram (ECG)

The PowerLab system (ADInstruments, Colorado Springs, CO) was utilized to measure and record the electrocardiograms (ECGs) of both sham and VO mice at P56.

### 2.14 Statistical analysis

Statistical analyses were performed using SAS software version 9.2 (SAS Institute Inc., Cary, NC, United States). Continuous data were expressed as means ± one standard deviation. We analyzed differences with Student’s t-test when the data were normally distributed; otherwise, data were tested with the Mann–Whitney test. *p* < 0.05 was considered to be statistically significant.

## 3 Results

### 3.1 Development of neonatal RA VO in mice with ACF surgery

**FIGURE 1:**
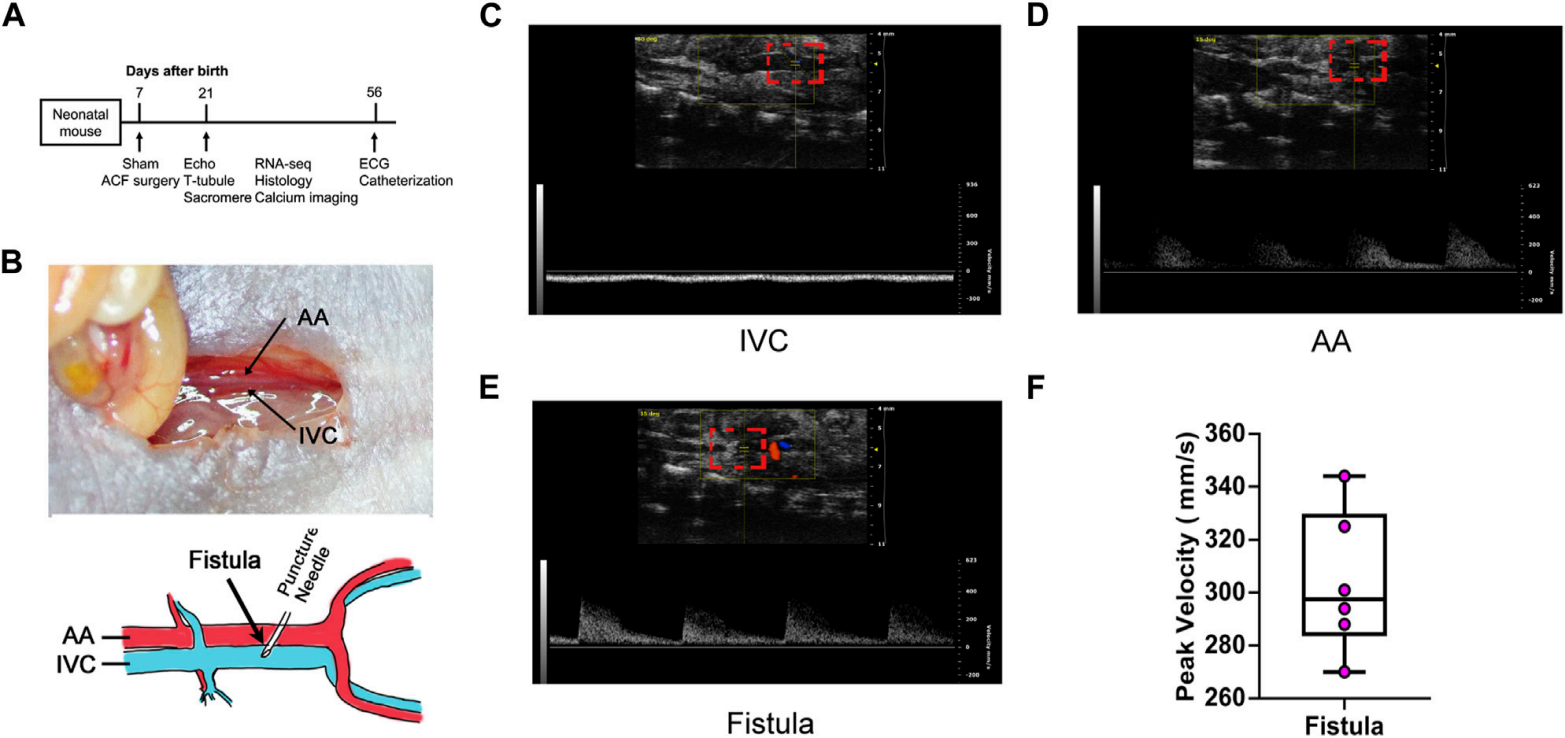
Abdominal aorta and inferior vena cava fistula creation and verification. **(A)** Timeline diagram of the experiment protocol. **(B)** Upper panel: Local anatomy of the abdominal aorta (AA) and inferior vena cava (IVC). Lower panel: Schematic of the fistula surgery. An illustration video can be found at: (https://www.ahajournals.org/doi/suppl/10.1161/JAHA.121.020854). **(C)** IVC manifested no pulsatile blood flow. **(D)** AA manifested pulsatile blood flow. **(E)** Fistula manifested pulsatile blood flow. **(F)** Quantification of the fistula peak velocity.

As shown in Figure 1A, we conducted ACF and sham procedures on P7 and performed analyses at P14 and P56. Because AA and IVC are adjacent to each other in the abdomen, puncturing the adjacent site of AA and IVC to create a fistula allows blood to directly flow from AA to IVC, thereby causing a substantial increase in the cardiac blood volume return (Figure 1B). Under normal physiological conditions, there is no pulsatile blood flow in IVC (Figure 1C), but there is pulsatile blood flow in AA (Figure 1D). At the fistula, a pulsatile blood flow should be noted to verify the successful creation of a fistula between AA and IVC (Figures 1E, F). Among 35 mice that underwent sham surgery, one died, representing a mortality rate of 2.86%, while two of the 35 mice that underwent VO surgery died, representing a mortality rate of 5.71%. The combined mortality rate for both groups is 4.29%. Out of the 33 surviving VO mice, 30 had a sustained shunt, resulting in a successful VO rate of 92.8%.

**FIGURE 2:**
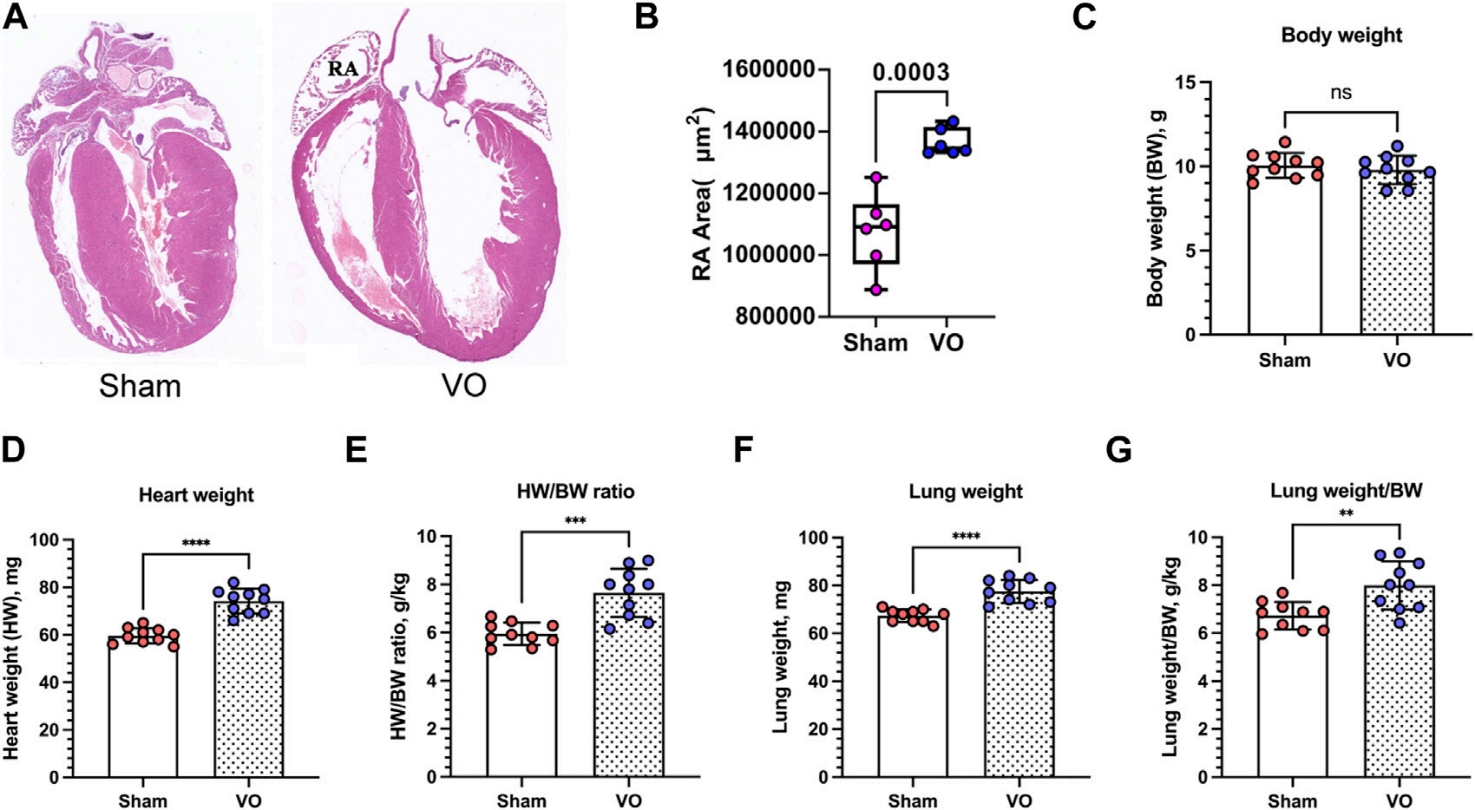
Verification of RA VO with H&E staining and organ weights. **(A)** Representative RA images. **(B)** Quantification of the RA area at the midpoint of the coronal section. **(C–G)** Quantification of body weight (BW), heart weight (HW), HW/BW ratio, lung weight, and lung weight/BW ratio in the sham and VO groups. *n* = 6, Student’s t-test. ns, no significance; **p < 0.01, ***p < 0.001, ****p < 0.0001.

To confirm that RA VO was induced, we examined the RA morphology and RA area at the midpoint of the coronal section. We found a larger RA with an increased RA area (Figures 2A, B). The heart/body weight ratios (HW/BW) and lung/body weight ratios (lung/BW) were significantly elevated in the VO group at P21 (Figures 2C–G; Table 1).

**TABLE 1:** Organ weight parameters.

Parameter	Sham (*n* = 10)	ACF (*n* = 10)	*p*-value
Body weight (BW), g	10.06 ± 0.7369	9.79 ± 0.8468	0.4617
Heart weight (HW), mg	59.6 ± 3.204	74.2 ± 5.181****	<0.0001
HW/BW ratio, g/kg	5.949 ± 0.4624	7.649 ± 1.005***	0.0001
Lungs, mg	67.3 ± 2.627	77.5 ± 4.79****	<0.0001
Lungs/BW, g/kg	6.725 ± 0.5736	7.988 ± 1.011**	0.003

Data are expressed as mean ± SD; *p < 0.05, **p < 0.01, ***p < 0.001, ****p < 0.0001.

Bold values are significantly differ between the two groups.

**FIGURE 3:**
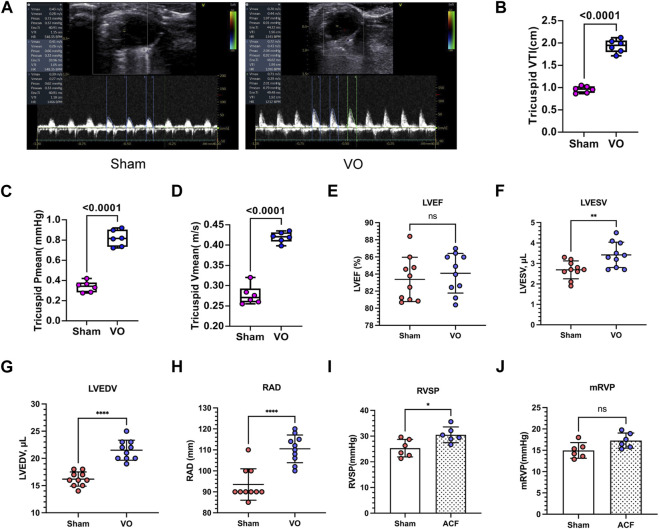
Verification of RA VO with echocardiography and cardiac catheterization. **(A)** Representative echocardiography at the tricuspid levels. Note the sharp waveform in the VO group. **(B)** Quantification of tricuspid mean velocity (Vmean). **(C)** Quantification of the tricuspid mean pressure gradient (Pmean). **(D)** Quantification of the tricuspid velocity time integral (VTI). **(E–H)** Quantification of left ventricular ejection fractions (LVEFs), left ventricular end-systolic volumes (LVESVs), left ventricular end-diastolic volumes (LVEDVs), and right atrium diameters (RADs) in the sham and VO groups. **(I, J)** Quantification of right ventricular systolic pressures (RVSPs) and mean right ventricular pressures (mRVPs) in the sham and VO groups. *n* = 6, Student’s t-test. ns, no significance; *p < 0.05, **p < 0.01, ****p < 0.0001.

To further verify RA VO, we examined the hemodynamic changes at the tricuspid valve. The results showed that the tricuspid mean velocities (tricuspid Vmean) in the sham and VO groups were 0.277 ± 0.024 and 0.419 ± 0.013 m/s, respectively (*p* < 0.0001, *n* = 6, Figures 3A, B); the tricuspid mean pressure (tricuspid Pmean) values in the sham and VO groups was 0.338 ± 0.054 and 0.818 ± 0.082 mmHg, respectively (*p* < 0.0001, *n* = 6, Figures 3A, C); and the tricuspid velocity time integral (VTI) values in the sham and VO groups were 0.958 ± 0.054 and 1.929 ± 0.149 cm, respectively (*p* < 0.0001, *n* = 6, Figures 3A, D).

To determine whether heart failure had occurred, we evaluated the function of the left ventricle. The results showed that the left ventricular ejection fractions (LVEFs) and left ventricular fractional shortenings (LVFSs) were not significantly influenced by VO at P21 (Figure 3E; Supplementary Figure S3A; Table 2). Nevertheless, the left ventricular end-systolic volumes (LVESVs), left ventricular end-diastolic volumes (LVEDVs), right atrium diameters (RADs), and stroke volumes (SVs) exhibited noticeable increases in response to VO conditions (Figures 3F–H; Supplementary Figure S3B; Table 2).

**TABLE 2:** Echocardiographic parameters.

Parameter	Sham (*n* = 10)	ACF (*n* = 10)	*p*-value
HR (bpm)	555.4 ± 16.26	551.1 ± 22.77	0.6315
LVEF (%)	83.37 ± 2.588	84.09 ± 2.322	0.5214
LVFS (%)	45.46 ± 1.034	45.05 ± 1.356	0.4559
LVESV, μL	2.69 ± 0.4383	3.42 ± 0.6197*	0.007
LVEDV, μL	16.2 ± 1.317	21.5 ± 1.841****	<0.0001
SV, μL	13.51 ± 1.215	18.08 ± 1.562****	<0.0001
RAD (mm)	93.5 ± 7.472	110.5 ± 6.587****	<0.0001

Data are expressed as mean ± SD; *p < 0.05, **p < 0.01, ***p < 0.001, ****p < 0.0001.

HR, heart rhythm; LVEF, left ventricular ejection fraction; LVFS, left ventricular fractional shortening; LVESV, left ventricular end-systolic volume; LVEDV, left ventricular end-diastolic volume; SV, stroke volume; RAD, right atrial diameter.

Bold values are significantly differ between the two groups.

Monitoring the hemodynamic status of the heart is essential for assessing the reliability of this model. We performed cardiac catheterization to measure pressures in the hearts of sham and VO mice. The results demonstrated a significant increase in RVSP under VO conditions, while no significant differences were observed in RVDP, mRVP, and the heart rate (HR) between the sham and VO groups (Figures 3I, J; Supplementary Figures S3C, D; Table 3).

**TABLE 3:** Hemodynamic parameters for sham and ACF groups at postnatal week 8.

Parameter	Sham (*n* = 6)	ACF (*n* = 6)
**RVSP, mmHg**	25.32 ± 3.41	30.52 ± 3.06*
**RVDP, mmHg**	4.633 ± 1.61	4.05 ± 1.06
**mRVP, mmHg**	14.98 ± 1.83	17.28 ± 1.78
**HR, beats/min**	565 ± 32.65	546 ± 22.2

Data are expressed as mean ± SD; *p < 0.05.

RVSP, right ventricular systolic pressure; RVDP, right ventricular diastolic pressure; mRVP, mean right ventricular pressure; HR, heart rate.

Bold values are significantly differ between the two groups.

The aforementioned morphologic and hemodynamic results suggested that a neonatal RA VO mouse model without heart failure was successfully induced by ACF surgery.

### 3.2 VO impedes RA cardiomyocyte sarcomere and T-tubule maturation

**FIGURE 4:**
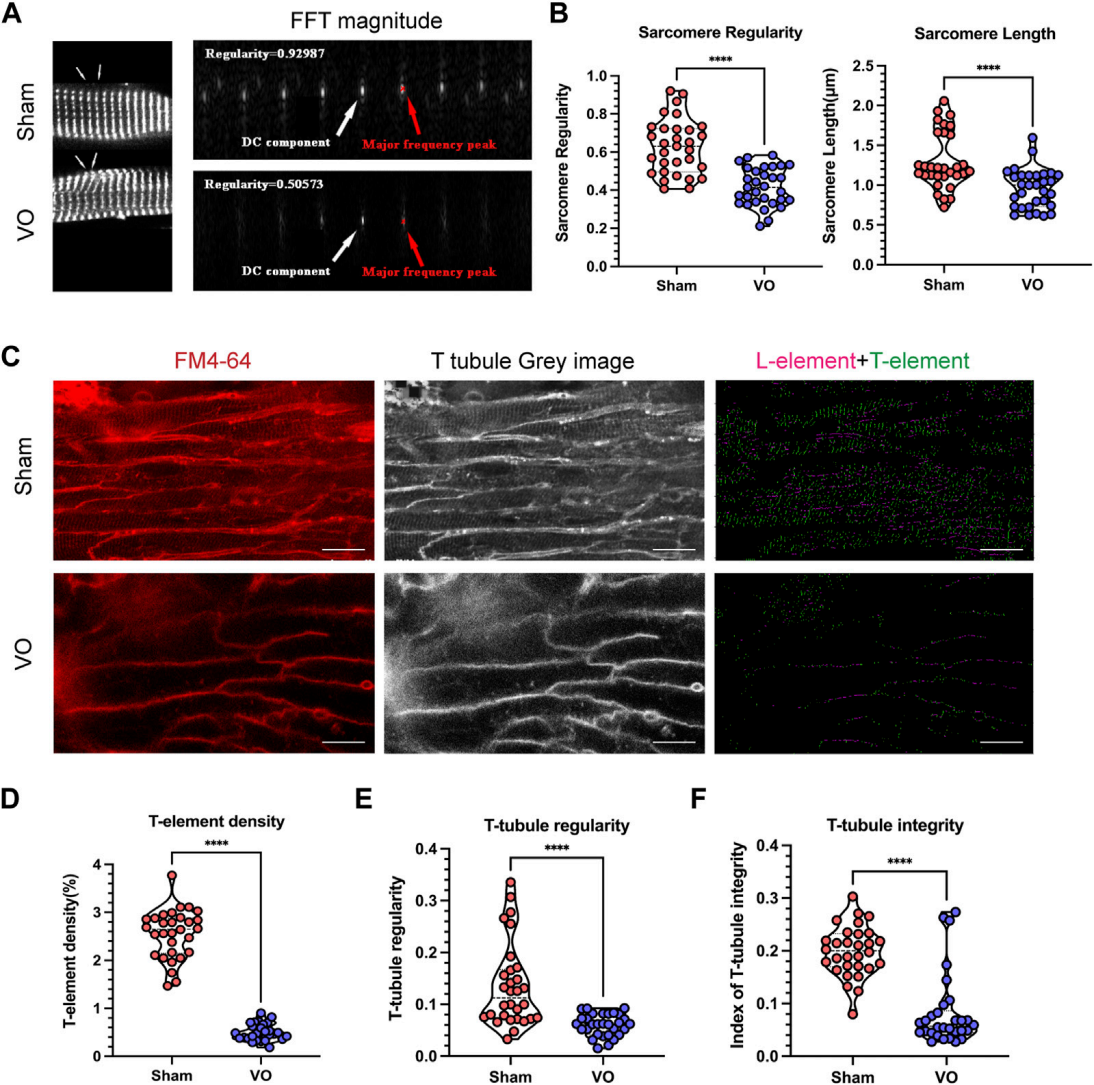
Impaired sarcomere and T-tubule maturation caused by VO. **(A)** Left panel: Representative sarcomere from the sham and VO groups. Sarcomeric α-actinin (SAA, white) staining, where arrow indicates one sarcomere. Right panel: Representative magnitude of fast Fourier transform (FFT) of cardiomyocytes shown in **(A)**; the direct current (DC) component was defined as the transformed series at frequency 0, which represents the summation of signals of all pixels in the image; the major frequency was defined as the second highest peak; regularity was defined as the magnitude of the major frequency normalized to that of the DC component. **(B)** Quantification of sarcomere regularity and sarcomere length from cardiomyocytes in each group (*n* = 30), Mann–Whitney test. **(C)** Representative T-tubule image. The T-element (green) is highlighted (right panel). Scale bar, 25 μm. **(D)** Quantification of the T-element density from cardiomyocytes in each group (*n* = 30), Mann–Whitney test. **(E)** Quantification of T-tubule regularity from cardiomyocytes in each group (*n* = 30), Mann–Whitney test. **(F)** Quantification of the index of T-tubule integrity from cardiomyocytes in each group (*n* = 30), Mann–Whitney test. ****p < 0.0001.

Because the sarcomere is a basic structure of cardiomyocytes, the maturation of which determines the electrophysiological function of cardiomyocytes (Stout et al., 2018; Brida et al., 2022), we first observed whether VO affected RA sarcomere maturation. With VO, the RA cardiomyocyte sarcomere regularity reduced from 0.628 ± 0.144 μm to 0.417 ± 0.099 μm, and the RA cardiomyocyte sarcomere length reduced from 1.297 ± 0.357 μm to 0.954 ± 0.244 (Figures 4A, B). These results suggested that VO impeded the maturation of RA cardiomyocyte sarcomeres.

T-tubules are characteristic markers that distinguish mature from immature cardiomyocytes. They are formed by the invagination of the myocardial cell membrane and serve as the structural basis for the coupling of excitation and contraction of cardiomyocytes (Perera et al., 2022; Tarasov et al., 2023). We then determined whether VO affected the T-tubule maturation of RA cardiomyocytes. The results showed that under VO conditions, the T-element density of RA cardiomyocytes was reduced from 2.552% ± 0.509% to 0.4978% ± 0.171%; the T-tubule regularity was reduced from 0.135 ± 0.080 to 0.060 ± 0.021; and the index of T-tubule integrity was reduced from 0.199 ± 0.048 to 0.083 ± 0.069 (Figures 4C–F). These results suggested that VO impeded the maturation of RA cardiomyocyte T-tubules.

### 3.3 VO impedes the electrophysiological activity of RA cardiomyocytes

**FIGURE 5:**
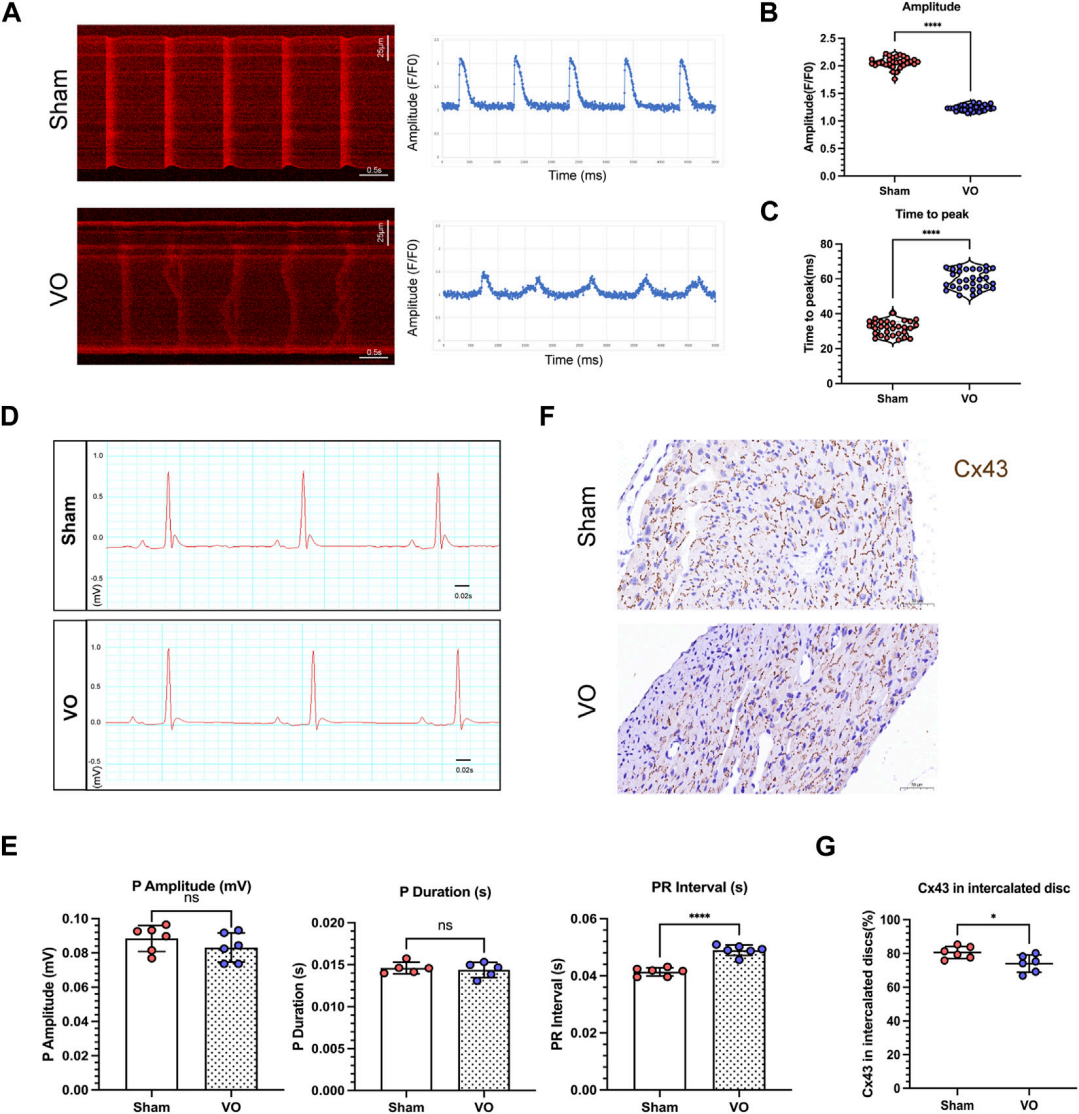
Impaired electrophysiological activity of RA cardiomyocytes caused by VO. **(A)** Left panel: Representative calcium transient image of RA cardiomyocytes from sham and VO mice. Right panel: Plot representative of the calcium transient image of RA cardiomyocytes from sham and VO mice. **(B)** Quantification of the calcium transient amplitude (Amp) from cardiomyocytes of three hearts in each group (*n* = 30), Mann–Whitney test. **(C)** Quantification of the calcium transient time to peak from cardiomyocytes of three hearts in each group (*n* = 30), Mann–Whitney test. **(D)** Representative electrocardiogram of sham and VO mice. **(E)** Quantification of the P-wave amplitude, P-wave duration, and PR interval in mice in each group (*n* = 6). **(F)** Representative IHC image of connexin 43 (Cx43) from sham and VO RA. **(G)** Quantification of the Cx43 percentage in intercalated discs in mice in each group (*n* = 6). ns, no significance; *p < 0.05, ****p < 0.0001.

The T-tubule system primarily coordinates the excitation–contraction coupling of cardiomyocytes and is characterized by maturation of the calcium-handling capability. Thus, we examined calcium transients in cardiomyocytes to further validate the results of impaired T-tubule maturation due to VO. The findings indicated that under the influence of VO, there was a significant reduction in the amplitude of cardiomyocyte calcium transients, accompanied by a notable prolongation of the time to peak (Figures 5A–C).

The atrial electrophysiological characteristics on the electrocardiogram were also analyzed, as shown in Figure 5D. The results showed that the PR intervals increased significantly in VO mice compared with the sham mice, with no significant differences in P-wave amplitude or duration (Figure 5E).

Immunohistochemistry showed that Cx43 became more lateralized with a significantly decreased Cx43 percentage in the intercalated discs in the VO group compared with the sham group (74.02% ± 5.14% in VO mice vs. 80.58% ± 3.64% in sham mice, *n* = 6 per group, *p* = 0.0286) (Figures 5F, G).

Sirius Red and Masson’s trichrome staining methods were used to evaluate fibrosis in the right atrium. As shown in Supplementary Figure S4, Sirius Red and Masson’s trichrome staining demonstrated minimal collagen fiber areas, with no significant differences observed between the sham and VO groups at P21. These results suggested that VO did not cause obvious fibrosis in RA at P21.

### 3.4 VO impedes the maturation of sarcomeres and T-tubules in RA cardiomyocytes at the transcriptome level

**FIGURE 6:**
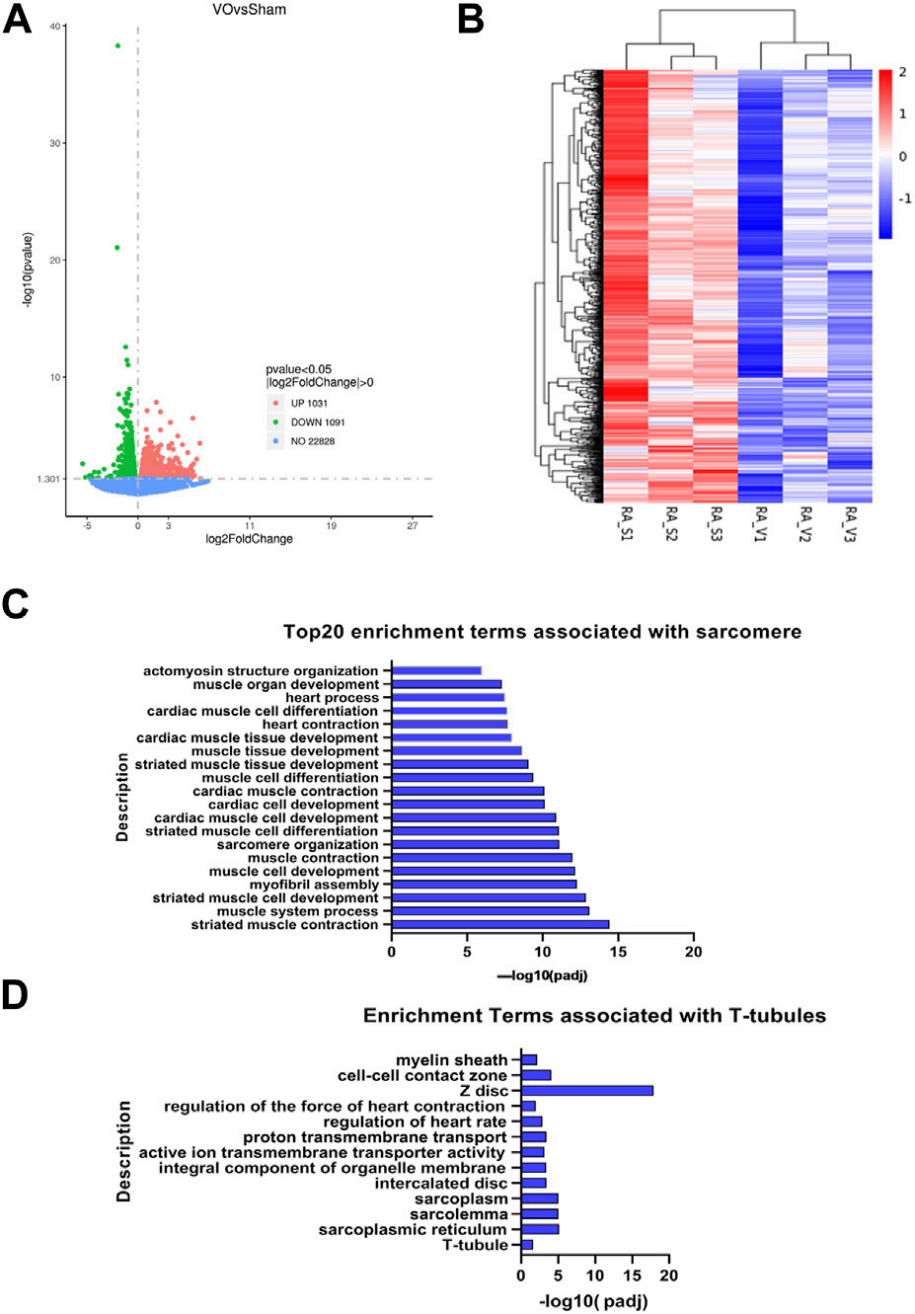
RNA-seq analysis of VO-induced downregulated genes in RA. **(A)** Volcano map of differentially expressed genes. Noted that there were 1,031 upregulated genes and 1,091 downregulated genes, which were then subjected to GO enrichment analysis and cluster analysis. **(B)** Cluster analysis of the 1,091 downregulated genes showed differences between groups and consistency within groups. **(C)** Top 20 enriched GO terms associated with sarcomeres. **(D)** Enriched GO terms associated with T-tubules.

To further confirm that the maturation of RA sarcomeres and T-tubules was impaired by VO, we then sought evidence at the transcriptome level. RNA-seq analysis revealed that there were 2,122 differentially expressed genes (DEGs) between the sham and VO RAs, with 1,031 upregulated and 1,091 downregulated genes (Figure 6A), suggesting that VO significantly altered the gene expression of RAs. We then subjected the downregulated DEGs to GO analysis. The cluster analysis of downregulated DEGs showed consistency within groups and differences between groups (Figure 6B). GO analysis revealed that there was an abundance of enriched terms associated with sarcomeres and T-tubules, which included striated muscle cell development, sarcomere organization, T-tubules, and sarcoplasmic reticulum (Figures 6C, D). These results further suggested that the maturation of sarcomeres and T-tubules in RA was impaired by VO.

### 3.5 Verification of RNA-seq results by qRT-PCR

To confirm the RNA-seq results, the sarcomere- and T-tubule-associated genes enriched in the GO terms were verified by qRT-PCR.

**FIGURE 7:**
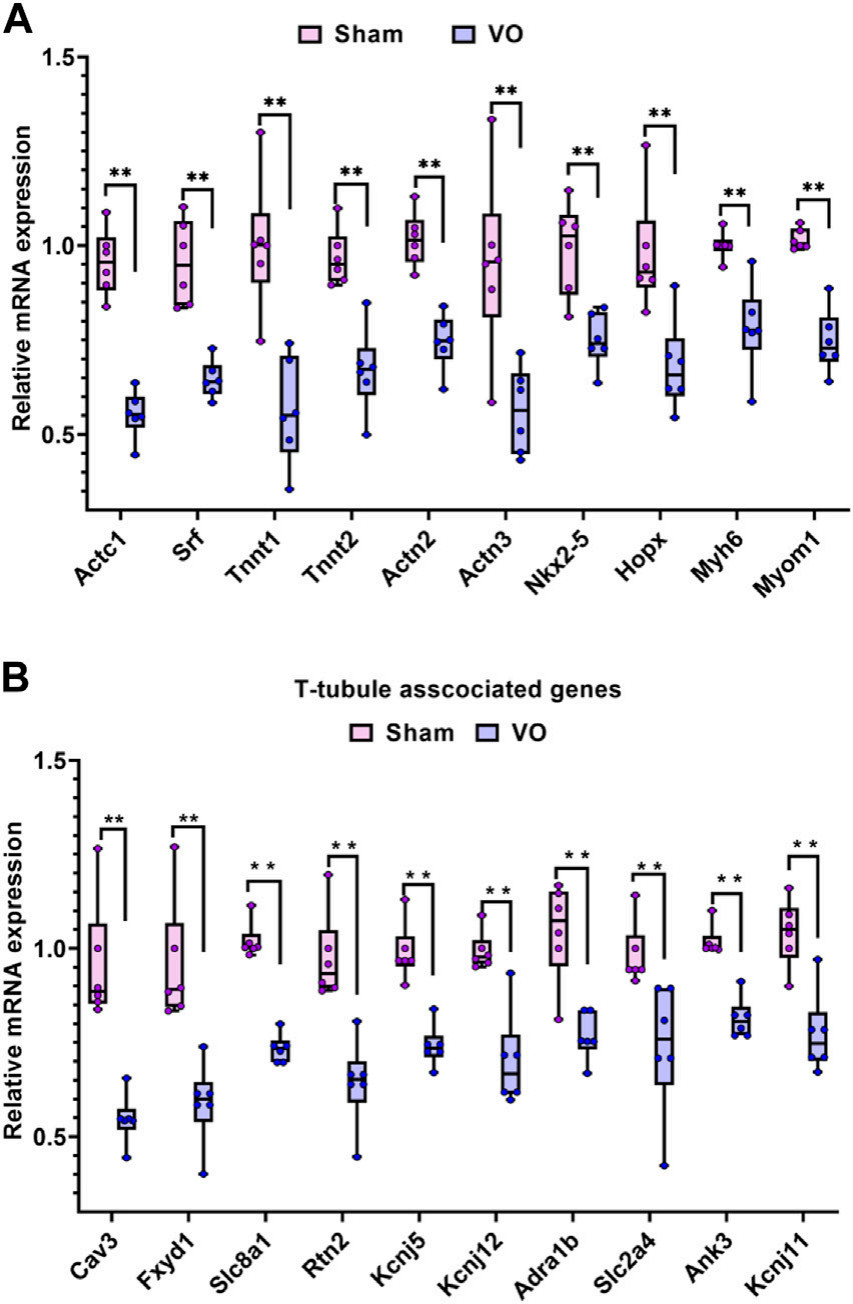
Verification of the sarcomere- and T-tubule-associated genes enriched in the GO analysis of downregulated genes. **(A)** Ten representative sarcomere-associated genes enriched in the GO term of striated muscle cell development. **(B)** Ten representative T-tubule-associated genes enriched in the GO term of T-tubules.

As shown in Figure 7A, the 10 sarcomere-associated genes under the GO term striated muscle cell development (*Actc1*, *srf*, *Tnnt1*, *Tnnt2*, *Actn2*, *Actn3*, *Nkx2-5*, *Hopx*, *Myh6*, and *Myom1*) were significantly downregulated in the VO group compared to the sham group. As shown in Figure 7B, 10 sarcomere-associated genes (*Cav3*, *Fxyd1*, *Slc8a1*, *Rtn2*, *Kcnj5*, *Kcnj12*, *Adra1b*, *Slc2a4*, *Ank3*, and *KcnJ11*) were significantly downregulated in the VO group compared to the sham group. The qRT-PCR results were consistent with RNA-seq enrichment analysis, suggesting an impairment of sarcomeres and T-tubules in RA because of VO.

## 4 Discussion

The most recent state-of-the-art review from the European Society of Cardiology (ESC) reported that atrial arrhythmias were relatively common in ASDs, and the prevalence of atrial arrhythmias in patients with ASD increased steeply with age, with up to 20% of the patients experiencing atrial flutter (Craig and Selzer, 1968; John Sutton et al., 1981; Berger et al., 1999; Ueda et al., 2013). The extent of RA structural remodeling is highly dependent on the duration of VO and is associated with frequent electrophysiological alterations that include reduced voltages, prolonged refractory periods, and conduction disturbances (Ueda et al., 2013; Stout et al., 2018; Brida et al., 2022). Moreover, the frequency of arrhythmias between early and delayed ASD closure differed (Berger et al., 1999). However, the ESC guideline for ASD only suggests that there should be a timely closure of ASD when a patient presents with right ventricular VO (Baumgartner et al., 2021). From the aspect of atrial cardiomyocyte maturation, the current study suggests that ASD should be closed as early as possible and may be a supplement to the ESC guideline for ASD closure.

Recently, cardiomyocyte maturation has attracted increased attention because it is a cornerstone for the clinical application of pluripotent stem cell (PSC)-derived cardiomyocytes, which are immature (Guo and Pu, 2020; Karbassi et al., 2020). Immature cardiomyocytes have high automaticity, characterized by high expression of pacemaker channels, a resting membrane potential close to the action potential activation threshold, and spontaneous Ca^2+^ release. Thus, immature cardiomyocytes transplanted into hearts *in vivo* easily caused arrhythmias (Chong et al., 2014). However, the mechanisms of regulating cardiomyocyte maturation are largely unknown, especially under pathological conditions such as VO and pressure overload, the two primary hemodynamic changes in CHD (Wang et al., 2017; Ye et al., 2020; Sun et al., 2021). The current study demonstrates that VO impedes the maturation of RA cardiomyocytes, characterized by immature sarcomere structures and underdeveloped T-tubule systems, resulting in impaired calcium-handling capacities, reduced levels of Cx43 in the intercalated discs, and prolonged PR intervals. These findings suggest that enhancing RA cardiomyocyte maturation may serve as a therapeutic target for preventing arrhythmias in cases of delayed ASD closure.

Sarcomeres play a critical role in cardiomyocyte maturation by organizing intracellular structures and modulating signal transduction (Guo et al., 2018; Guo et al., 2021). For example, mutation of the sarcomere gene *Actn2* leads to the defective structural maturation of T-tubules and mitochondria and perturbs the nuclear localization of the SRF cofactor MRTFA, a critical transcription factor that regulates cardiomyocyte maturation (Guo et al., 2021). Our results showed that VO downregulated Actn2 and SRF in RA cardiomyocytes (Figure 7A), suggesting that VO may impede RA cardiomyocyte maturation via the sarcomere gene *Actn2* and transcription factor SRF.

The limitation to the current pioneering study is that the molecular mechanisms through which VO impedes RA cardiomyocyte maturation were not explored. As mechanical stress, VO requires mechanical receptors to transmit mechanical signals to cardiomyocytes. Then, what are the mechanical receptors? As previously reported, of three mechanical receptors, Sdc4, Itga11, and Plxnd1 (Mehta et al., 2020; Romaine et al., 2022), only *Sdc4* was included in the upregulated gene list (Supplementary Figure S1). It is possible that Sdc4 mediates the effects of VO on RA cardiomyocyte maturation. However, this hypothesis requires further research to verify its reliability. Previous studies suggested that immune responses played an important role in ventricular responses to VO (Cui et al., 2021; Sun et al., 2021; Hu et al., 2022). We also found that many enriched GO terms of the upregulated genes were associated with immune responses (Supplementary Figure S2). It has been reported that after activating the mechanical receptor Plxnd1 on endothelial cells, the endothelial cells upregulated the expression of monocyte chemotactic factor MCP-1 and vascular adhesion factor VCAM1, which recruits and activates macrophages (Mehta et al., 2020). Macrophages then regulate the maturation of cardiomyocytes through paracrine factors (Lavine et al., 2014; Li et al., 2020). Thus, it is possible that VO activates Sdc4 on endothelial cells which then recruit immune cells to regulate RA cardiomyocyte maturation.

In summary, the current study was the first to introduce a neonatal mouse model of RA VO. By using the model, we demonstrated that VO impeded the sarcomere and T-tubule maturation of RA cardiomyocytes, resulting in impaired calcium-handling capacity, reduced connexin levels, and prolonged PR intervals. Thus, we suggest that the immaturity of RA cardiomyocytes may account for arrhythmias in adult patients with ASD.

## Data Availability

The datasets presented in this study can be found in online repositories. The names of the repository/repositories and accession number(s) can be found below: https://www.ncbi.nlm.nih.gov/geo/; GSE232594.

## Supplementary material

SUPPLEMENTARY FIGURE S1 Fold change of mechanoreceptor Sdc4.

SUPPLEMENTARY FIGURE S2 Top 20 enriched terms of the GO analysis of the upregulated genes.

SUPPLEMENTARY FIGURE S3 Additional data from echocardiography and cardiac catheterization in sham and VO mice. **(A,B)** Quantification of left ventricular fractional shortenings (LVFSs) and stroke volumes (SVs) in sham and VO groups. *n* = 6, Student’s t-test. **(C,D)** Quantification of right ventricular diastolic pressures (RVDPs) and heart rates (HRs) in sham and VO groups at P56. *n* = 6, Student’s t-test. ns, no significance; **p* < 0.05, ***p* < 0.01, *****p* < 0.0001.

SUPPLEMENTARY FIGURE S4 VO did not cause significant RA fibrosis. **(A)** Representative Masson’s trichrome and Sirius Red staining of RA sections from sham and VO mice. **(B)** Quantification of the collagen fiber area percentage in mice in each group. *n* = 6; ns, no significance.
