# Supplementary material for: Volume overload impedes the maturation of sarcomeres and T-tubules in the right atria: a potential cause of atrial arrhythmia following delayed atrial septal defect closure
Source: Front Physiol. 2023 Oct 16;14:1237187. doi: 10.3389/fphys.2023.1237187 (PMC10614073; doi:10.3389/fphys.2023.1237187)
Supplement: Supplementary file 1 [file Table1.DOCX]

Supplemental Table 1 Primer information

| Gene |  | Sequence (5'->3') |
| --- | --- | --- |
| Actc1 | Forward | CTGGATTCTGGCGATGGTGTA |
|  | Reverse | CGGACAATTTCACGTTCAGCA |
| Srf | Forward | GGCCGCGTGAAGATCAAGAT |
|  | Reverse | CACATGGCCTGTCTCACTGG |
| Tnnt1 | Forward | AAGGGGAGCGTGTGGATTTTG |
|  | Reverse | TCCTCCTTTTTCCGCTGTTCA |
| Tnnt2 | Forward | CAGAGGAGGCCAACGTAGAAG |
|  | Reverse | CTCCATCGGGGATCTTGGGT |
| Actn2 | Forward | TGGCACCCAGATCGAGAAC |
|  | Reverse | GTGGAACCGCATTTTTCCCC |
| Actn3 | Forward | AACAGCAGCGGAAAACCTTCA |
|  | Reverse | GGCTTTATTGACATTGGCGATTT |
| Nkx2-5 | Forward | GACAAAGCCGAGACGGATGG |
|  | Reverse | CTGTCGCTTGCACTTGTAGC |
| Hopx | Forward | GACCGTCCCCAACCTGACTA |
|  | Reverse | CAACTTGTGCAGTGAGCGTC |
| Myh6 | Forward | GCCCAGTACCTCCGAAAGTC |
|  | Reverse | GCCTTAACATACTCCTCCTTGTC |
| Myom1 | Forward | GCACGACCATGAGCCACTAC |
|  | Reverse | ACCCTTGAGAATGCCGGGA |
| Cav3 | Forward | GGATCTGGAAGCTCGGATCAT |
|  | Reverse | TCCGCAATCACGTCTTCAAAAT |
| Fxyd1 | Forward | TCCATTCACCTACGATTACCACA |
|  | Reverse | GAATTTGCATCGACATCTCTTGC |
| Slc8a1 | Forward | CTTCCCTGTTTGTGCTCCTGT |
|  | Reverse | AGAAGCCCTTTATGTGGCAGTA |
| Rtn2 | Forward | AGTGGCAGACCTGTTGTACTG |
|  | Reverse | ACGGACACGATGCTAAAGTGC |
| Kcnj5 | Forward | GCCGGTGATTCTAGGAATGCT |
|  | Reverse | TCCTGAACATTACCGTGGTGT |
| Kcnj12 | Forward | ACCCCTACAGCATCGTATCAT |
|  | Reverse | GTTGCACTGACCGTTCTTCTT |
| Adra1b | Forward | CGGACGCCAACCAACTACTT |
|  | Reverse | AACACAGGACATCAACCGCTG |
| Slc2a4 | Forward | GTGACTGGAACACTGGTCCTA |
|  | Reverse | CCAGCCACGTTGCATTGTAG |
| Ank3 | Forward | GGCCTTACCCCAATCCATGTT |
|  | Reverse | TCCTTAGCTTTTGCTTCTACCTG |
| Kcnj11 | Forward | AAGGGCATTATCCCTGAGGAA |
|  | Reverse | TTGCCTTTCTTGGACACGAAG |
