# Supplementary figures and images for: Volume overload impedes the maturation of sarcomeres and T-tubules in the right atria: a potential cause of atrial arrhythmia following delayed atrial septal defect closure
Source: Front Physiol. 2023 Oct 16;14:1237187. doi: 10.3389/fphys.2023.1237187 (PMC10614073; doi:10.3389/fphys.2023.1237187)

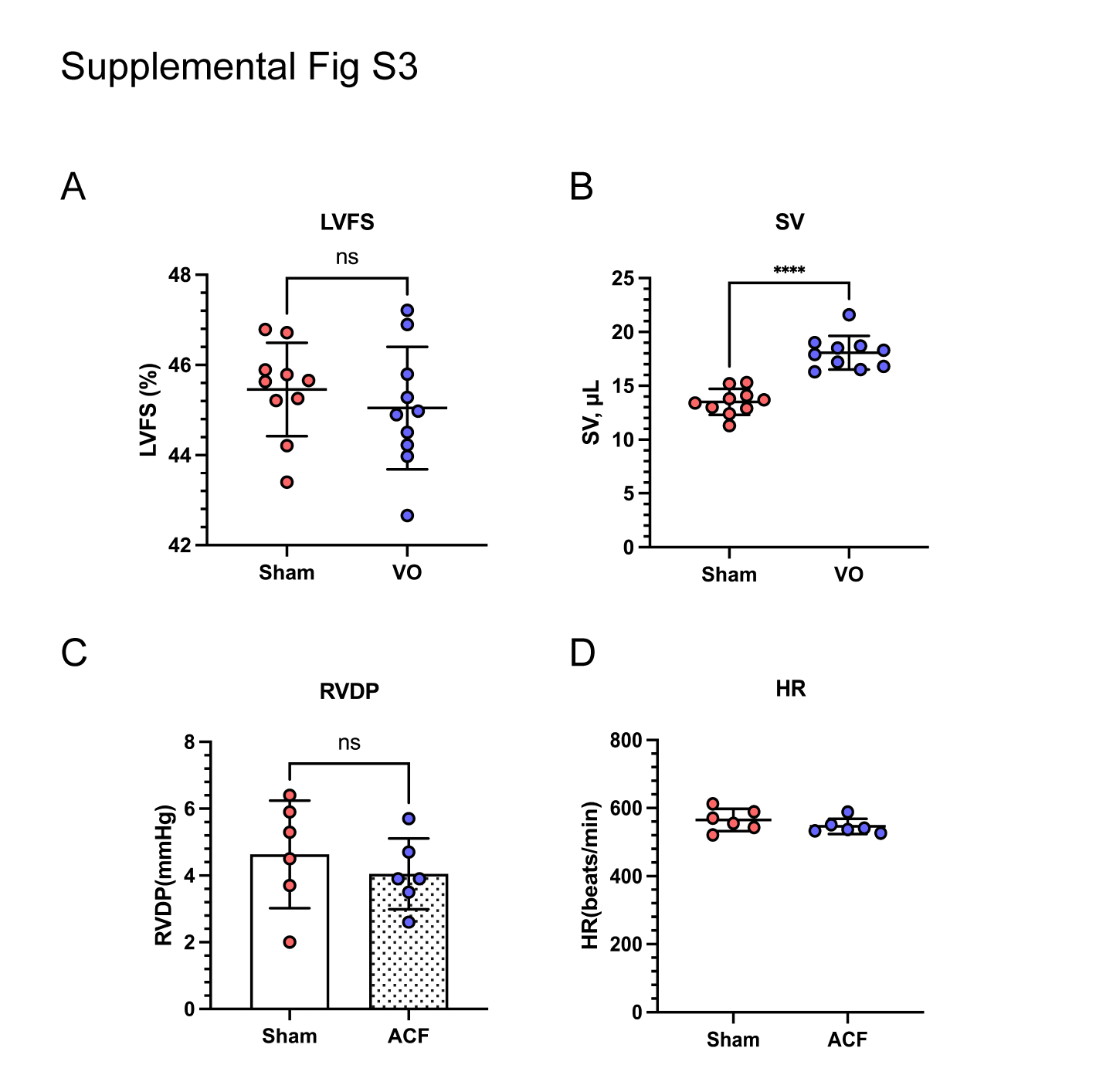

Supplement: Supplementary file 2 [file Image3.TIF]

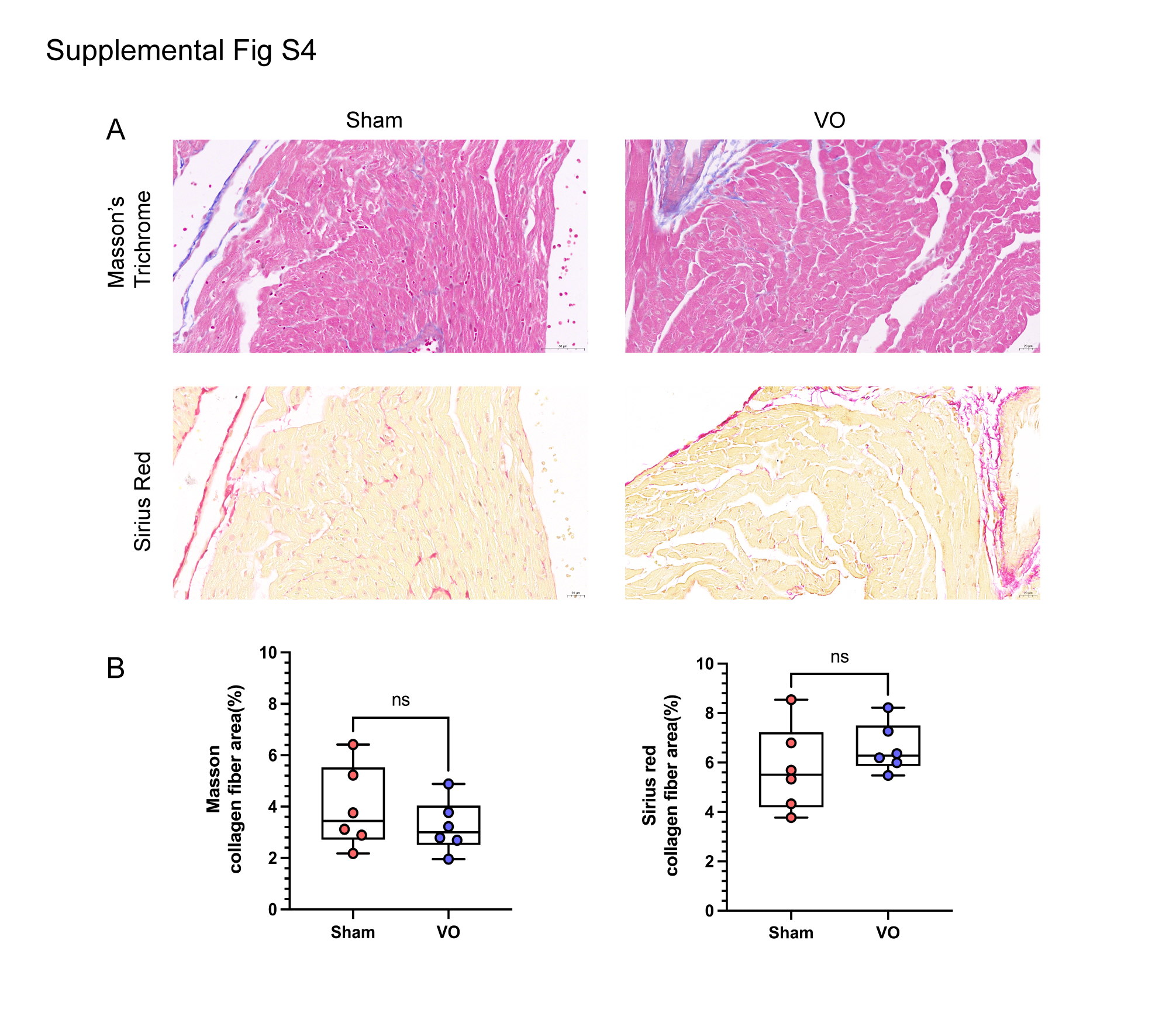

Supplement: Supplementary file 3 [file Image4.TIF]

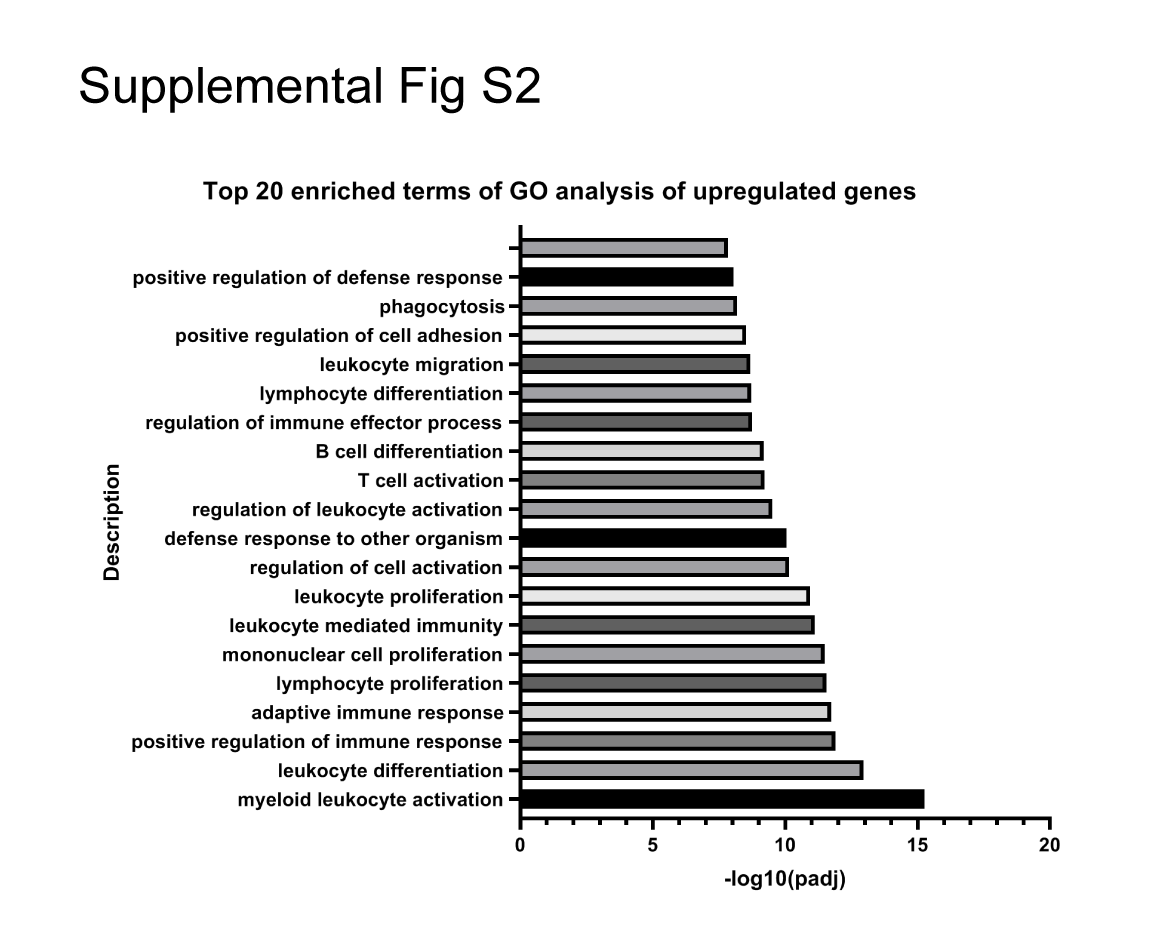

Supplement: Supplementary file 4 [file Image2.TIF]

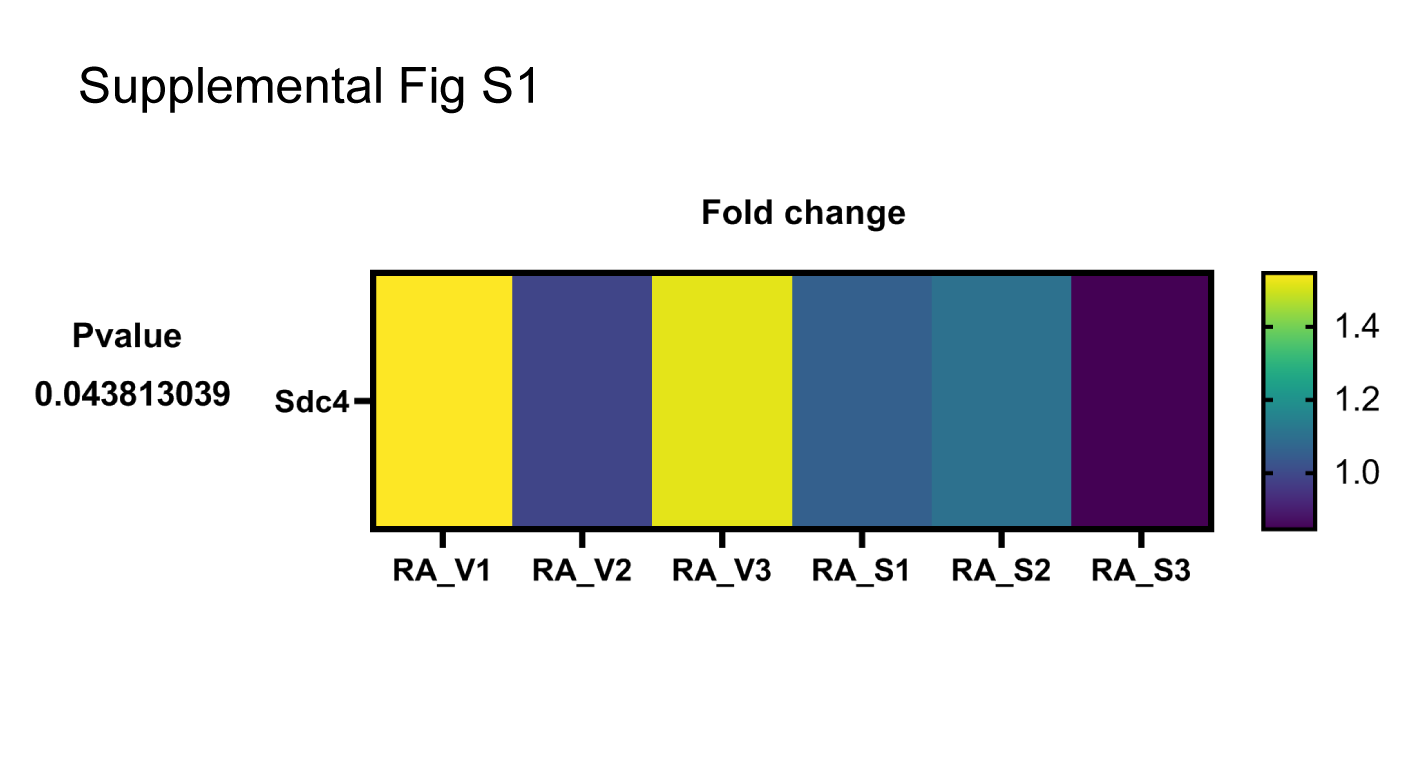

Supplement: Supplementary file 5 [file Image1.TIF]
