# Supplementary material for: Volume overload impedes the maturation of sarcomeres and T-tubules in the right atria: a potential cause of atrial arrhythmia following delayed atrial septal defect closure
Source: Front Physiol. 2023 Oct 16;14:1237187. doi: 10.3389/fphys.2023.1237187 (PMC10614073; doi:10.3389/fphys.2023.1237187)
Supplement: Supplementary file 6 [file Table2.DOCX]

Supplemental Table 2 Reagents

| Name | Company | Catalog No. |
| --- | --- | --- |
| Triton X-100 | Sigma-Aldrich | T9284 |
| Hematoxylin and eosin Kit | Beyotime biotech | C0105M |
| DNase | Worthington, Lakewood, NJ, USA | 9003-98-9 |
| RNase | Worthington, Lakewood, NJ, USA | 9001-99-4 |
| paraformaldehyde(PFA) | Sigma-Aldrich | 158127 |
| 4',6-diamidino-2-phenylindole(DAPI) | ThermoFisher Scientific | D3571 |
| Rhod-4™, AM | AAT bioquest | 21121 |
| MM4-64 | AAT bioquest | 21487 |
| Type II collagenase | Worthington | 9001-12-1 |
| PureLink RNA Micro Scale Kit | Life Technologies, Carlsbad, California, USA | 12183016 |
| PrimeScriptTM reagent kit | Takara Bio, Kusatsu, Japan | RR037A |
| SYBR Green Power Premix Kits | Applied Biosystems, Foster City, California | 4368577 |
| NEB Next® UltraTM RNA Library Prep Kit | NEB, USA | E7760 |
| TruSeq PE Cluster Kit | Illumina | v3-cBot-HS |
